# Influence of Initial Yield Strength Weighting on Residual Stresses in Quenched Cylinders Using Finite Element Analysis

**DOI:** 10.3390/ma17235833

**Published:** 2024-11-28

**Authors:** Junpeng Li, Yingqiang Xu, Youwei Liu

**Affiliations:** School of Mechanical Engineering, Northwestern Polytechnical University, Xi’an 710072, China; geneli@mail.nwpu.edu.cn (J.L.); you-wei.liu@mail.nwpu.edu.cn (Y.L.)

**Keywords:** quenching, residual stress, initial yield strength, medium-carbon steel, finite element models

## Abstract

Using the quenching process to create a specific residual stress distribution in steel parts is a key method for improving their strength. Although finite element simulation can overcome the time-consuming and labor-intensive limitations of experimental measurements, accurately predicting the residual stress distribution in quenched steel parts remains a challenge for researchers and manufacturers. The initial yield strength weighting scheme used in finite element simulations has a significant impact on the results. To investigate the influence of initial yield strength weighting on the residual stress distribution in quenched steel cylinders, finite element models with different yield strength weightings have been developed. The results show that the large hardness difference between austenite and martensite can cause significant deviations between the residual stress predicted using linear weighting and the experimental results. The linear weighting scheme commonly used by researchers overestimates the yield strength of the austenite phase in the mixed-phase material during cooling, leading to an overestimation of residual stress. Employing nonlinear yield strength weightings, such as Leblond weighting, can significantly improve the computational accuracy of finite element models, yielding more reliable and consistent predictions. This improved accuracy in predicting residual stress using finite element simulation offers a powerful tool for optimizing the quenching process.

## 1. Introduction

With the rapid development of industry, carbon steel is widely used in various engineering applications due to its high strength, toughness, and cost-effectiveness [1,2]. Quenched and tempered AISI 4140 steel is extensively used in manufacturing transmission components, such as marine crankshafts and gears [3]. During the quenching process, surface hardness is enhanced by the formation of a martensitic microstructure, which is promoted by a rapid cooling rate [4,5,6]. Additionally, the development of residual compressive stresses on the surface after quenching can further increase the material’s strength [7,8]. Advances in computational technology have enabled the use of finite element simulations to predict residual stress distributions in quenched parts, providing a more efficient alternative to traditional experimental methods [9,10]. Several commercial finite element software programs, such as DEFORM [11,12], DANTE [13,14], SYSWELD [15], and ABAQUS [16,17], can perform heat treatment simulations.

**Figure 1:**
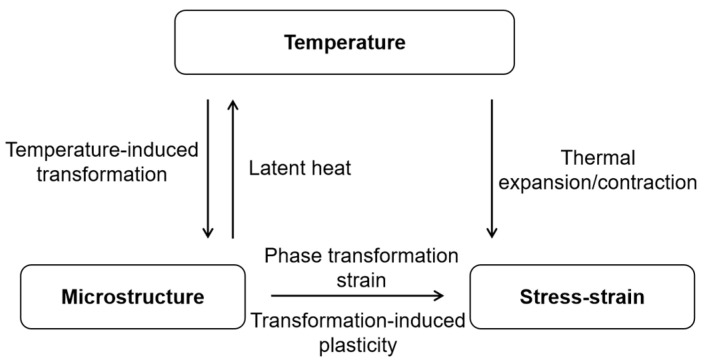
Simplified coupling relationship of quenching process.

While finite element simulations offer significant advantages over conventional quenching experiments, their accuracy in predicting residual stress distribution is highly dependent on the precision of the underlying material models, particularly the constitutive model [16]. Quenching is a complex process in which temperature, microstructure, and stress/strain fields are intricately coupled. The simplified coupling relationship is illustrated in Figure 1. Research into finite element simulations of the quenching process spans nearly half a century. Early experimental studies by Leblond et al. [18] helped establish a consensus that transformation-induced plasticity (TRIP) must be included in the material’s constitutive model [9,16,19]. While models incorporating transformation-induced plasticity are reasonably accurate in predicting residual stress, studies have shown that the initial yield strength weighting scheme significantly impacts the calculating results [20]. The commonly used linear weighting scheme often leads to deviations between calculated and experimental residual stress results [20]. Other weighting schemes, such as the Geijselaers [20] and Leblond [18] weightings, are less frequently employed. Most commercial finite element software typically defaults to a linear weighting scheme and does not allow for the input of alternative schemes. Furthermore, few studies have explored how different initial yield strength weightings affect residual stress distributions after quenching.

Therefore, this study aims to address this gap by investigating the influence of various initial yield strength weightings on residual stress. In this work, we developed finite element models incorporating four different initial yield strength weighting schemes to simulate the quenching process. The effects of these weightings on the residual stress distribution in a quenched cylinder were compared. A modified K-M model was proposed for the martensitic phase transformation kinetics, and the transformation temperature was based on a thermodynamics model developed earlier [21]. The quenching test was conducted on an AISI 4140 steel cylinder with a diameter of 50 mm and a length of 150 mm. Temperature changes on the surface were recorded using a K-type thermocouple positioned 1 mm below the surface, and X-ray diffraction (XRD) was employed to measure the residual stress distribution along the radial direction. The cooling curves obtained from finite element simulations were compared to experimental results, and the residual stress distributions, calculated using different initial yield strength weightings, were also compared with experimental measurements. Finally, the impact of different initial yield strength weightings on the residual stress distribution after quenching was analyzed.

## 2. Materials and Methods

### 2.1. Experimental Methods

The composition of the studied steel, AISI 4140, obtained through chemical analysis, is listed in Table 1. The dimensions of the cylinder used to verify the simulation model are 50 mm in diameter and 150 mm in length. A K-type thermocouple was inserted 1 mm below the surface of the cylinder to measure the cooling curves during quenching.

The sample was initially heated to 850 °C (1123 K) in a pit furnace. To ensure complete austenitization, it was held at this temperature for 90 min. Subsequently, the sample was transferred and immersed in 18 °C (291 K) water for 5 min until its temperature dropped below 50 °C (323 K).

Residual stresses in the quenched cylinder were measured using an X-ray stress instrument (iXRD, PROTO, Waterloo, ON, Canada) with CrK_α_ radiation (wavelength of 0.2291 nm), operating at 20 kV and 4 mA. The method used was the $\sin^{2}\psi$ method [22]. The measurement was performed using the iso-inclination method, with a spot diameter of 2 mm and the Fe{211} diffraction surface. Ψ angles were set to ±0°, ±12°, ±24°, ±30°, ±37°, and ±43°.

The residual stress distribution along the depth direction of the cylinder was determined by sequentially turning the outer layer and etching with a 15% nitric acid aqueous solution in 0.5 mm layers. It should be noted that removing the outer layer may induce redistribution of residual stress, which was corrected using Equations (1) and (2) [23].

(1) $$\sigma_{t}\left(r\right)=\sigma_{tm}\left(r\right)-\int_{r}^{R}\sigma_{tm}\left(\xi \right)\cdot \frac{d\xi }{\xi },$$

(2) $$\sigma_{a}\left(r\right)=\sigma_{am}\left(r\right)-2\int_{r}^{R}\sigma_{am}\left(\xi \right)\cdot \frac{d\xi }{\xi }.$$

where $\sigma_{t}\left(r\right)$ and $\sigma_{a}\left(r\right)$ represent the corrected tangential and axial residual stresses at radius *r*, respectively, while $\sigma_{tm}\left(r\right)$ and $\sigma_{am}\left(r\right)$ denote the measured tangential and axial residual stresses at radius *r*, respectively. *R* is the initial radius of the specimen (25 mm).

### 2.2. Modeling Approach

#### 2.2.1. Temperature Distribution

When calculating the temperature field during quenching, it is crucial to consider the influence of latent heat of phase transformations to ensure accuracy. Compared to temperature changes caused by heat conduction, the energy associated with strain deformation has a negligible effect on the temperature field change (about 2 K) [24]. This study disregards the impact of strain deformation energy on the temperature field. The governing equation for computing the temperature field based on transient Fourier’s law is shown as follows:

(3) $$\rho C_{p}\dot{T}=\nabla \left(k\cdot \nabla T\right)+\dot{Q}.$$

where ρ is the material density, obtained by linearly weighting the densities of each phase. $C_{p}\;\mathrm{and}\;k$ are the specific heat capacity at constant pressure and thermal conductivity of the material, respectively, which vary with temperature and phase composition. Specific heat capacity and thermal conductivity data for each phase of AISI 4140 used in this study are adopted from Kakhki et al. [25]. *T* represents temperature. $\dot{T}$ denotes the time derivative of temperature. ∇ represents the gradient operator. $\dot{Q}$ is the sum of the latent heat rate of each phase transformation calculated using Equation (4).

(4) $$\dot{Q}=\sum_{j=2}^{3}\Delta H_{k}{\dot{\phi }}_{k}.$$

where $\Delta H_{k}$ is the enthalpy change when the *k*-th phase transformation occurs. ${\dot{\phi }}_{k}$ is the transformation rate of the *k*-th phase, representing the rate of change in the phase fraction of bainite (*k* = 2) and martensite (*k* = 3) with time. The enthalpy changes for the transformation from austenite to bainite and martensite are $-5.12\times 10^{8}\;\mathrm{and}\;-3.14\times 10^{8}\;J/m^{3}$, respectively.

Assuming complete austenitization, the initial temperature field analysis sets the temperature to 850 °C. A film boundary condition is applied at the surface where the cylinder contacts the quenching medium:

(5) $$-k\nabla T=h\left(T\right)\left(T_{s}-T_{m}\right).$$

where *h*(*T*) is the heat transfer coefficient of the quenching medium, dependent on the surface temperature *T*_s_ of the cylinder and the temperature *T*_m_ of the quenching medium (291 K or 18 °C).

**Figure 2:**
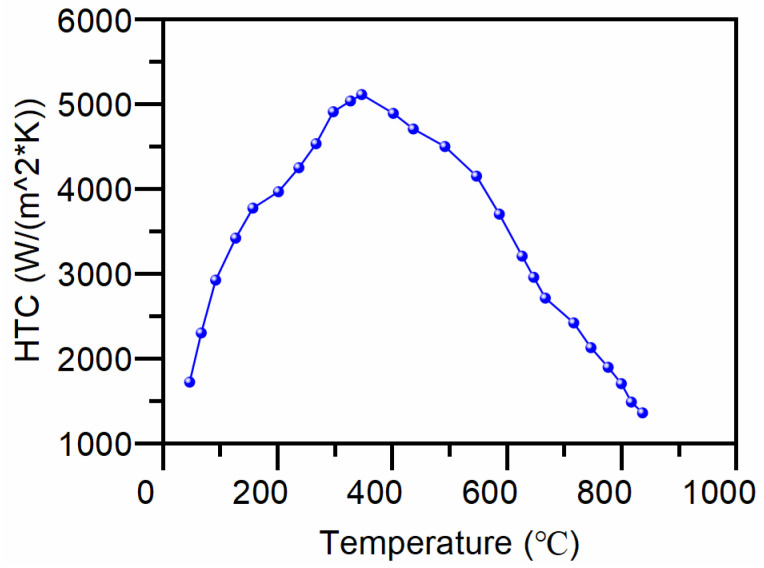
Heat transfer coefficient (HTC) computed using the inverse heat transfer method.

After measuring the surface cooling curves with a thermocouple, an inverse heat transfer algorithm is employed to determine the heat transfer coefficient as a function of temperature. Linear interpolation is then utilized to estimate the remaining temperature values. Figure 2 illustrates the heat transfer coefficient curve computed using the inverse heat transfer method.

#### 2.2.2. Phase Transformation Kinetics

**Figure 3:**
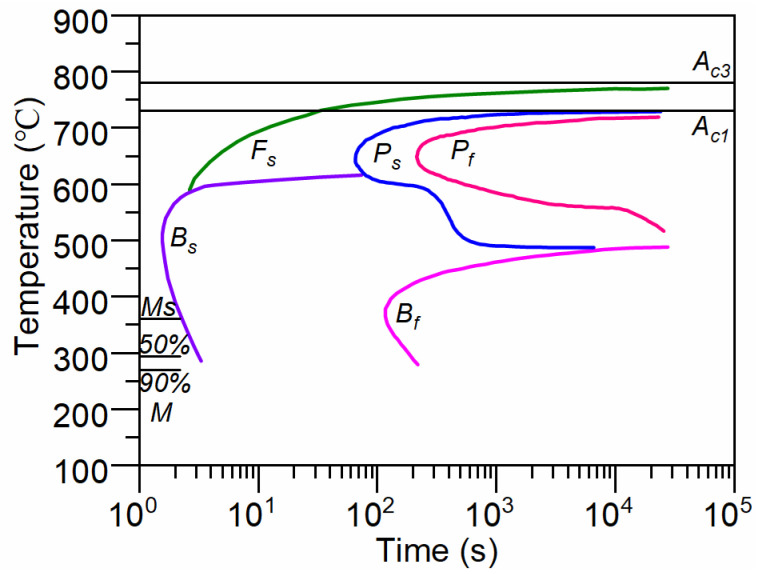
TTT diagram of AISI 4140 [26].

Figure 3 illustrates the isothermal transformation diagram (TTT diagram) of AISI 4140 steel (Fe-0.38C-0.64Mn-0.23Si-0.99Cr-0.16Mo-0.08Ni) [26]. Due to slight variation in chemical composition compared to the steel used in the literature to establish the TTT diagram, corrections were applied to the TTT curves in this study. The correction parameters adjusted include *M*_s_, *B*_s_ and the overall TTT curve, as referenced in the literature [27]. From the cooling curves of the cylinder, it is evident that the slowest cooling rate at the core of the cylinder is 15 K/s. Based on the corrected TTT curves, we conclude that for AISI 4140 steel bars with a diameter of 50 mm, there is no transformation into ferrite and pearlite during quenching. Consequently, the phase transformation model only needs to calculate the phase fractions of bainite and martensite.

When the temperature drops below *B*_s_, bainite transformation initiates. This study adopts the empirical formula proposed by Lee [28] to calculate the upper limit of bainite transformation temperature *B*_s_:

(6) $$B_{s}\left({}^{\circ}C\right)=745-110C-59\mathrm{Mn}-39\mathrm{Ni}-68\mathrm{Cr}-106\mathrm{Mo}+17\mathrm{MnNi}+6{\mathrm{Cr}}^{2}+29{\mathrm{Mo}}^{2}$$

Bainitic transformation is a diffusion-type phase transformation. The Johnson–Mehl–Avrami–Kolmogorov (JMAK) model [29], coupled with the additive rule, is extensively used for calculating diffusion-type transformations. However, the JMAK model does not fully account for the gradual cessation characteristics at the end of bainite transformation [30]. Upon cooling to the bainite start temperature, the transformation proceeds at the following rate:

(7) $$\frac{dX}{dt}=\frac{2^{\left(G-1\right)/2}\cdot \Delta T^{2}\cdot \exp\left[-\frac{115,115}{RT\left(t\right)}\right]\cdot X^{2\left(1-X\right)/3}\cdot {\left(1-X\right)}^{2X/3}}{\left(2.34+10.1\%C+3.8\%\mathrm{Cr}+19\%\mathrm{Mo}\right)\cdot 10^{-4}\cdot f\left(X,C_{i}\right)},$$

where

(8) $$f\left(X,C_{i}\right)=\exp\left[X^{2}\cdot \left(1.9\%C+2.5\%\mathrm{Mn}+0.9\%\mathrm{Ni}+1.7\%\mathrm{Cr}+4\%\mathrm{Mo}-2.6\right)\right].$$

In the rate form of the equation, undercooling Δ*T* is defined as the difference between the current temperature and the temperature at which the bainite transformation begins (*B*_s_).

When the temperature is below *M*_s_, martensitic transformation occurs independently of time. Lee [31] has reviewed several empirical models for the kinetics of non-thermal martensitic transformation. Among these, the K-M equation proposed by Koistinen and Marburger [32] is widely utilized:

(9) $$\begin{array}{r} \xi_{M}=1-\exp\left[-0.011\cdot \left(M_{s}-T_{q}\right)\right]\\ M_{S}>T_{q}>193K\;\left(-80^{\circ }C\right) \end{array}.$$

where $\xi_{M}$ represents the volume fraction of martensite, and *T*_q_ denotes the lowest temperature reached during quenching.

**Figure 4:**
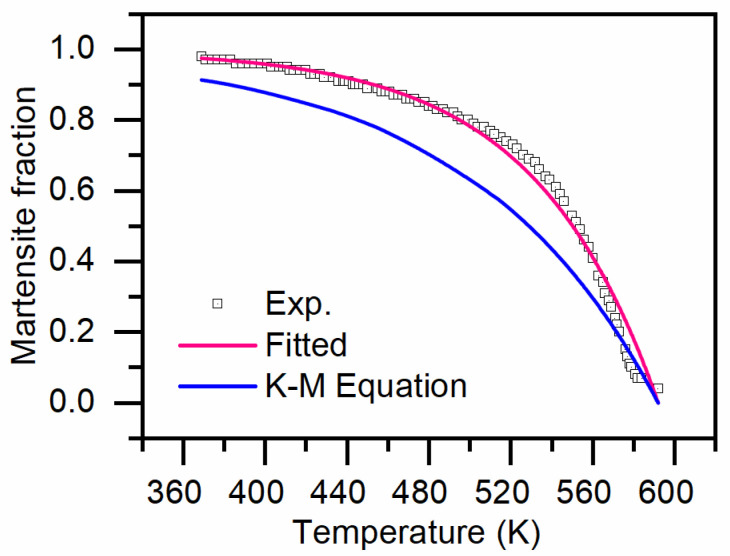
Kinetic model for martensitic transformation.

The equation above is derived from Fe-C alloys. However, when applying it to model the martensitic transformation kinetics of AISI 4140, which contains additional alloying elements, inaccuracies arise. To address this, we propose a modified K-M model that incorporates the influence of these alloying elements:

(10) $$\phi_{M}=1-\exp\left[-K_{L}\times \left(M_{s}-T\right)\right].$$

where *K*_L_ is a function of the chemical composition. The specific value of *K*_L_ is determined by fitting experimental data on martensitic transformation, as illustrated in Figure 4. For AISI 4140 investigated in this study, the fitted value of *K*_L_ is 0.01663. Figure 3 clearly demonstrates significant differences between the volume fraction of martensitic transformation calculated using the original K-M equation and experimental results for AISI 4140. The value of *M*_s_ is calculated using a thermodynamic-based model proposed by us [21].

#### 2.2.3. Analysis of Stress/Displacement

Since the quenching process occurs rapidly, the total strain increment can be decomposed into five components, excluding the creep effect:

(11) $$\Delta \varepsilon_{ij}=\Delta \varepsilon_{ij}^{\mathrm{el}}+\Delta \varepsilon_{ij}^{\mathrm{pl}}+\Delta \varepsilon_{ij}^{\mathrm{th}}+\Delta \varepsilon_{ij}^{\mathrm{tr}}+\Delta \varepsilon_{ij}^{\mathrm{trip}}.$$

where $\Delta \varepsilon_{ij}^{\mathrm{el}},\;\Delta \varepsilon_{ij}^{\mathrm{pl}},\;\Delta \varepsilon_{ij}^{\mathrm{th}},\;\Delta \varepsilon_{ij}^{\mathrm{tr}}\;\mathrm{and}\;\Delta \varepsilon_{ij}^{\mathrm{trip}}$ represent increments of elastic strain, plastic strain, thermal strain, phase transformation strain and transformation induced plasticity strain, respectively.

Thermal strain and phase transformation strain are isotropic and are calculated using the following formulas [33]:

(12) $$\Delta \varepsilon_{ij}^{\mathrm{th}}=\delta_{ij}\sum_{k=1}^{3}\xi_{k}\alpha_{k}\Delta T,$$

(13) $$\Delta \varepsilon_{ij}^{\mathrm{tr}}=\delta_{ij}\sum_{k=2}^{3}\Delta \xi_{k}\beta_{k}.$$

where *k* = 1, 2, 3 denote austenite, bainite and martensite phases, respectively; *α_k_* is the thermal expansion coefficient (TEC) of the *k*-th phase; *β_k_* is the expansion coefficient of the *k*-th phase transformation; *δ_ij_* is the Kronecker delta. The experimental data for the thermal expansion coefficients of each phase are provided in Table 2.

The stress increment is calculated using the following equation [34]:

(14) $$\begin{array}{r} \Delta \sigma_{ij}=\lambda \delta_{ij}\Delta \varepsilon_{kk}^{\mathrm{el}}+2\mu \Delta \varepsilon_{ij}^{\mathrm{el}}+\Delta \lambda \delta_{ij}\Delta \varepsilon_{kk}^{\mathrm{el}}+2\Delta \mu \varepsilon_{ij}^{\mathrm{el}}\\ \lambda =\frac{E}{1-2\nu }-\frac{E}{3\left(1+\nu \right)}\\ \mu =\frac{E}{2\left(1+\nu \right)} \end{array}.$$

where *σ_ij_* is the Cauchy stress tensor; *E* and *v* are the elastic modulus and Poisson’s ratio of the material, respectively, calculated using a linear weighting scheme of each phase.

This study assumes that the material’s deformation behavior is rate-independent. The plastic behavior adheres to the Von Mises yield criterion with isotropic hardening:

(15) $$\sqrt{\frac{3}{2}\left(s_{ij}-\alpha_{ij}\right)\left(s_{ij}-\alpha_{ij}\right)}-\sigma_{y}=0.$$

where *s_ij_* is the deviatoric stress tensor, *α_ij_* is the deviatoric back stress tensor, and *σ_y_* is the yield strength of the mixed phase.

Many scholars [18,19,35,36,37] use the linear mixture law to calculate the yield stress of a mixture of phases, which is accurate when all coexisting phases have comparable hardness. However, the phase transformation from austenite to martensite dominates the final stress distribution, involving phases with significantly different hardness. The yield stress of the martensite phase is often an order of magnitude higher than that of austenite, making the linear mixture law inappropriate. To explore the influence of different initial yield strength weighting scheme on the final residual stress distribution, this study designs various weighting schemes of initial yield strength:

1. Linear weighting scheme;
2. Geijselaers weighting scheme for martensite phase with austenite as the soft phase;
3. Geijselaers weighting scheme for both bainite and martensite with austenite as the soft phase;
4. Linear weighting scheme for martensite and bainite followed by Leblond weighting scheme for a mixture of martensite, bainite and austenite.

The weighting schemes are expressed as follows:

(16) $$\begin{array}{r} \left(a\right)\;\sigma_{y}=\xi^{\gamma }\sigma_{y}^{\gamma }+\xi^{b}\sigma_{y}^{b}+\xi^{m}\sigma_{y}^{m}\\ \left(b\right)\;\sigma_{y}=\xi^{\gamma }\sigma_{y}^{\gamma }+\xi^{b}\sigma_{y}^{b}+f\left(\xi^{m}\right)\sigma_{y}^{m}\\ \left(c\right)\;\sigma_{y}=\xi^{\gamma }\sigma_{y}^{\gamma }+f\left(\xi^{b}\right)\sigma_{y}^{b}+f\left(\xi^{m}\right)\sigma_{y}^{m}\\ \left(d\right)\;\sigma_{y}=g\left(\xi^{\gamma }\right)\sigma_{y}^{\gamma }+\left[1-g\left(\xi^{\gamma }\right)\right]\sigma_{y}^{\mathrm{bm}} \end{array},$$

where $\xi^{\gamma }$, $\xi^{b}$ and $\xi^{m}$ are phase fractions of austenite, bainite and martensite, respectively; $\sigma_{y}^{\gamma }$, $\sigma_{y}^{b}$ and $\sigma_{y}^{m}$ are initial yield strength of austenite, bainite and martensite, respectively; $\sigma_{y}^{\mathrm{bm}}$ is the yield strength of the mixed microstructure of bainite and martensite. In (b) and (c), $f\left(\xi^{b}\right)\;\mathrm{and}\;f\left(\xi^{m}\right)$ are:

(17) $$\begin{array}{r} f\left(\xi^{b}\right)=\xi^{b}\left(C+2\left(1-C\right)\xi^{b}-\left(1-C\right){\left(\xi^{b}\right)}^{2}\right)\\ C=1.383\frac{\sigma_{y}^{\gamma }}{\sigma_{y}^{b}} \end{array},$$

(18) $$\begin{array}{r} f\left(\xi^{m}\right)=\xi^{m}\left(C+2\left(1-C\right)\xi^{m}-\left(1-C\right){\left(\xi^{m}\right)}^{2}\right)\\ C=1.383\frac{\sigma_{y}^{\gamma }}{\sigma_{y}^{m}} \end{array}.$$

where in (d), $g\left(\xi^{\gamma }\right)$ is a normalized function of the phase fraction of austenite, with its value taken from reference [38].

The equivalent stress is initially calculated based on purely elastic behavior. If the predicted elastic stress exceeds the initial yield strength of the mixed microstructure, plastic flow occurs. In such cases, the backward Euler method integrates Equations (14) and (15).

Plastic strain in metals typically results from deviatoric stresses exceeding the material’s yield strength. However, during phase transformation, plastic deformation occurs even at lower external stress levels, termed phase-transformation-induced plastic strain (TRIP). Unlike classical plasticity, phase-transformation-induced plastic strain does not involve a yield criterion, and the stress levels that cause phase-transformation-induced plasticity is not sufficient to cause classical plastic strain even in the softest austenite phase [30]. The incremental form of phase-transformation-induced plastic strain [30] is:

(19) $$\Delta \varepsilon_{ij}^{\mathrm{trip}}=\frac{3}{2}Kf^{\prime }\left(\xi \right)h\left(\sigma_{\mathrm{eq}},\sigma_{y}\right)s_{ij}\Delta \xi .$$

where *K* is the phase transformation induced plasticity coefficient, $f^{\prime }\left(\xi \right)$ is the derivative of the normalized saturation function $f\left(\xi \right)$ with respect to the transformed phase fraction, $h\left(\sigma_{\mathrm{eq}},\sigma_{y}\right)$ is a function of the equivalent stress and the yield stress of the mixed microstructure. $h\left(\sigma_{\mathrm{eq}},\sigma_{y}\right)$ is introduced to consider the nonlinear effects caused by applied stress. The expression proposed by Leblond [30] is used in this study:

(20) $$h\left(\sigma_{\mathrm{eq}},\sigma_{y}\right)=\left\{\begin{array}{l} 1,\mathrm{if}\frac{\sigma_{\mathrm{eq}}}{\sigma_{y}}\leq 0.5\\ 1+3.5\left(\frac{\sigma_{\mathrm{eq}}}{\sigma_{y}}-0.5\right),\mathrm{if}\frac{\sigma_{\mathrm{eq}}}{\sigma_{y}}\geq 0.5 \end{array}\right..$$

The value of the phase transformation plasticity coefficient *K* is determined through experiments or theoretical calculations and depends on the type of phase transformation, potentially related to carbon content and temperature. Several models for the normalized saturation function [39] have been proposed, with commonly used models by Abrassart, Desalos, Leblond and Tanaka shown in Table 3.

Taleb [39] highlighted that the saturation function proposed by Abrassart and Desalos significantly underestimated experimental results. Therefore, the normalized saturation function proposed by Leblond is adopted in this study.

## 3. Results and Discussion

### 3.1. Cooling Curves

**Figure 5:**
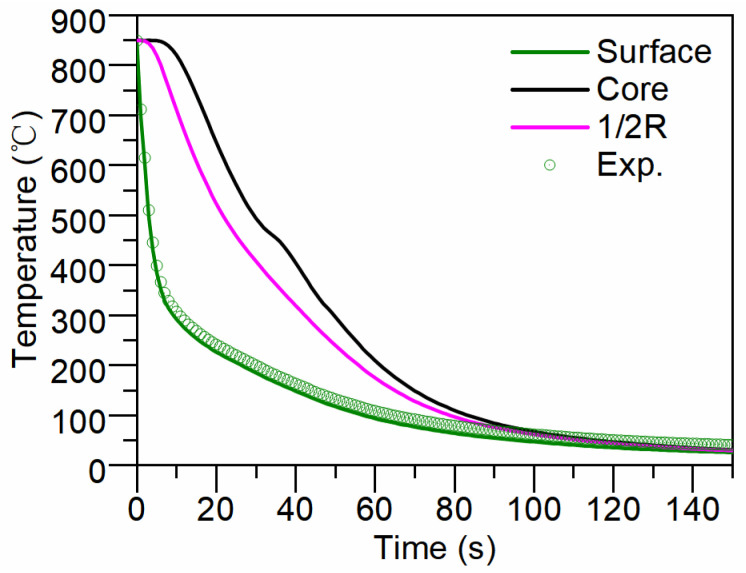
Calculated and measured cooling curves.

Figure 5 displays the calculated cooling curves. As depicted in Figure 5, the characteristic cooling rates (from 800 °C to 500 °C) at the surface, core and 1/2R (half-radius) of the cylinder are 113.6 K/s, 16.7 K/s and 19.1 K/s, respectively. It can be observed in Figure 5 that the “black” curve appears distorted. The distortion of the core’s cooling curve, observed between 30 and 40 s, is influenced by the latent heat of phase transformation. During the transformation from austenite to bainite or martensite, latent heat is released, which slows the cooling rate in the core. This phenomenon is not observed at the surface or at the 1/2 radius position, primarily due to the very rapid cooling rates at these locations, rendering the effect of the released latent heat negligible. As can be seen in Figure 5, the calculated cooling curve of surface is in good agreement with the experimental results.

### 3.2. Microstructure Distribution After Quenching

**Figure 6:**
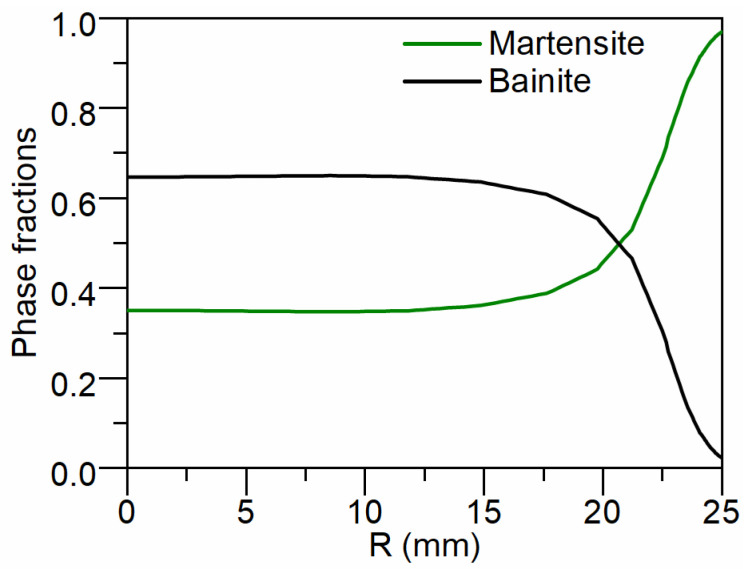
Calculated results of martensite and bainite distribution along the radius after quenching.

Figure 6 presents the calculated distributions of martensite and bainite along the radius after quenching. The fraction of martensite gradually decreases from the surface to the core. Specifically, the martensite fractions on the surface, 1/2R (half-radius) and the core are 97%, 35.2% and 35%, respectively. Conversely, the fraction of bainite increases from the surface to the core. The bainite fractions on the surface, 1/2R and the core are 2.3%, 64.5% and 64.7%, respectively.

### 3.3. Residual Stress Distribution After Quenching

**Figure 7:**
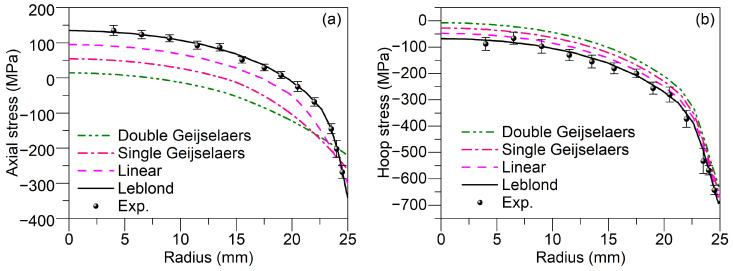
Residual stress distribution after quenching calculated using different weighting schemes compared with experimental results: (**a**) Axial stress; (**b**) Hoop stress.

Figure 7 depicts the comparison of calculated and experimental residual stress distributions from the surface to the core of the cylinder after quenching. Figure 7a,b show the axial and hoop residual stress distribution, respectively. The experimental results shown in the figure represent the average of two measurements, demonstrating good measurement accuracy is achieved as indicated by the error bars showing the standard deviation. From Figure 7, it is evident that the axial and hoop residual stress distribution calculated by the finite element model using the Leblond weighting scheme aligns best with the experimental results. Additionally, as shown in Figure 7a, the maximum tensile stress occurs at the core of the cylinder, while the maximum compressive stress is observed at the surface. Subsequently, we analyzed how various initial yield strength weighting schemes affect the distribution of hoop residual stress. As illustrated in Figure 7b, the hoop residual stress along the radial direction of the cylinder is compressive from the surface to the core, with the maximum compressive stress occurring at the surface and the minimum compressive stress at the core. The absolute values of stress amplitudes calculated using different yield stress weighting schemes range from smallest to largest as follows: double Geijselaers scheme, single Geijselaers scheme, linear scheme, and Leblond scheme. The primary reason for these variations lies in the Geijselaers weighting scheme, which tends to overestimate the contribution of austenite yield stress in multiphase material, thus underestimating the initial yield strength of the multiphase material. Consequently, the cylinder yields and undergoes plastic deformation at lower stress levels during quenching. This plastic deformation leads to stress relaxation, resulting in smaller calculated residual stress amplitudes. Conversely, compared to the linear weighting scheme, the Leblond weighting scheme reduces the proportion of austenite yield stress in the material, thereby providing a higher estimate of the initial yield strength of the material. Consequently, the calculated residual stress amplitudes are larger.

A coefficient (*CE*) is used to assess how closely the calculated residual stresses align with the experimental residual stresses. The coefficient is defined as:

(21) $$CE=\frac{1}{N}\cdot \sum \left(\frac{\left|\sigma_{\exp}-\sigma_{\mathrm{cal}}\right|}{\left|\sigma_{\exp}\right|}\right).$$

where *N* represents the number of experimentally measured stress locations, $\sigma_{\exp}$ represents the measured residual stress, and $\sigma_{\mathrm{cal}}$ denotes the calculated residual stress. Here, the value of the coefficient *CE* represents the average deviation between the measured and calculated residual stresses. The closer the value of *CE* is to zero, the better the agreement between the calculated and experimental results.

**Figure 8:**
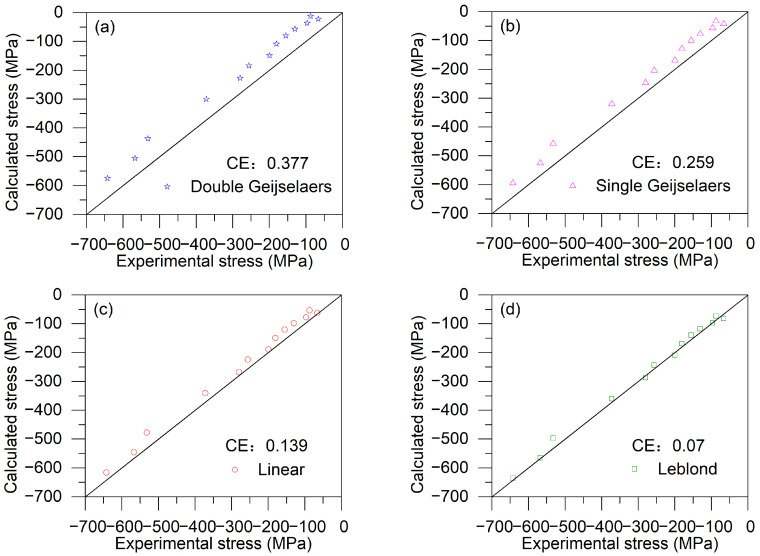
A comparison of the experimental results for residual stresses with the calculated results using different yield strength weighting schemes: (**a**) Double Geijselaers weighting scheme; (**b**) Single Geijselaers weighting scheme; (**c**) Linear weighting scheme; (**d**) Leblond weighting scheme.

Figure 8 compares the calculated residual stresses, obtained using different yield strength weighting schemes, with the experimental values. The *CE* values for the residual stress, calculated using the double Geijselaers, single Geijselaers, linear, and Leblond weighting schemes, are 0.377, 0.259, 0.139, and 0.07, respectively. As shown in Figure 8d, the residual stresses calculated using the Leblond weighting scheme exhibit the smallest *CE* value. The smallest *CE* value indicates that the finite element model using Leblond weighting scheme performs better and more accurately predicts the residual stress distribution after quenching.

**Figure 9:**
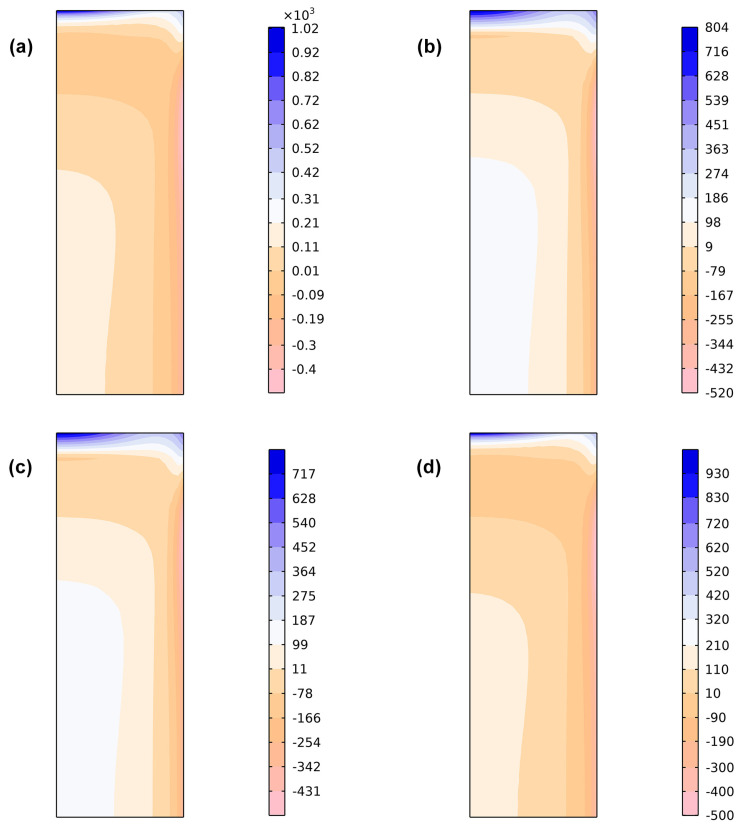
Axial residual stress contours of finite element simulations with different initial yield strength weightings: (**a**) Linear weighting scheme; (**b**) Single Geijselaers weighting scheme; (**c**) Double Geijselaers weighting scheme; (**d**) Leblond weighting scheme.

Figure 9 shows the distribution of axial residual stress obtained from finite element simulations using different initial yield stress weighting schemes. Subfigures (a), (b), (c) and (d) represent the results for linear weighting, single Geijselaers weighting, double Geijselaers weighting and Leblond weighting, respectively. It can be seen from Figure 9 that the maximum tensile stress occurs at the center of the top surface of the cylinder, with the position for linear weighting shifting slightly to the right. The maximum compressive stress occurs on the outer surface of the cylinder, approximately 20 mm from the top surface. The maximum residual tensile stress amplitude is greatest for linear weighting, surpassing that of Leblond weighting. The residual tensile stress amplitude is smallest for Geijselaers weighting, with single Geijselaers and double Geijselaers weightings showing similar results. Conversely, the residual compressive stress amplitude is smallest for the linear weighting scheme, smaller than that for Leblond weighting, while the largest amplitude occurs with Geijselaers weighting. Therefore, while different weightings have little effect on the locations of the maximum tensile and compressive residual stresses, they significantly influence the amplitudes of these stresses.

**Figure 10:**
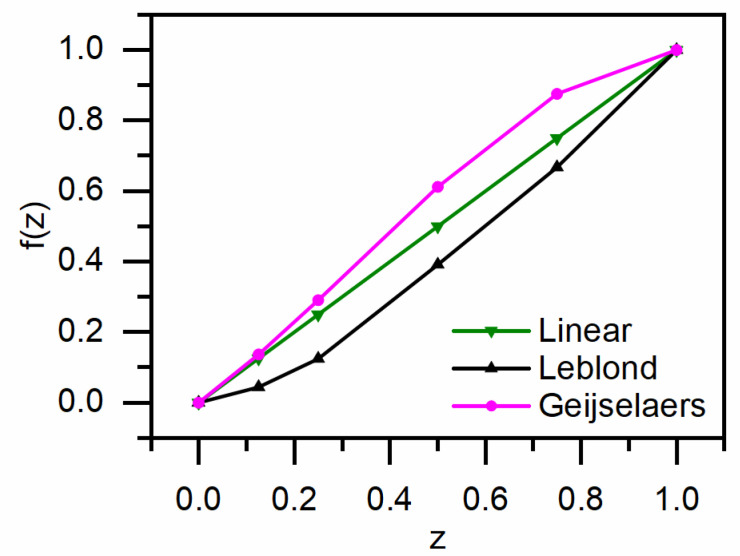
Proportion of austenite yield stress under various weighting schemes.

The accurate prediction of residual stress distribution relies on precise descriptions of temperature distribution, microstructure distribution, and constitutive relations. Different yield strength weighting schemes do not affect the calculation results of temperature and microstructure distributions. Therefore, the primary reason for differences in residual stress distribution lies in the variation in constitutive relations under different weighting schemes. The elasto-plastic model is crucial in stress calculations because classical plastic strain and phase-transformation-induced strain during quenching significantly influence stress distribution. A key physical parameter in the elasto-plastic model is the initial yield strength, which varies with temperature and phase composition. In materials undergoing phase transformation, multiple phases coexist. Many researchers typically employ a linear weighting scheme to construct constitutive models for initial yield strength. However, in multi-phase states, particularly since bainite and martensite have higher hardness than austenite, especially martensite, the use of a linear weighting scheme often results in an underestimation of the initial yield strength. This study examines the disparity in initial yield strength caused by different yield strength weighting schemes. Figure 10 illustrates the normalized function depicting the relationship between the austenite phase fraction and its weights of initial yield strength in the multi-phase material under various yield strength weighting schemes. On the horizontal axis, z represents the austenite phase fraction, and f(z) denotes the normalized function of the weights of initial yield strength for austenite. As observed in Figure 10, the Geijselaers weighting scheme exhibits a disproportionately higher proportion of austenite yield stress compared to the linear weighting scheme, which is evidently unrealistic. Conversely, the Leblond weighting scheme reduces the relative contribution of austenite yield strength within the mixed microstructure.

**Figure 11:**
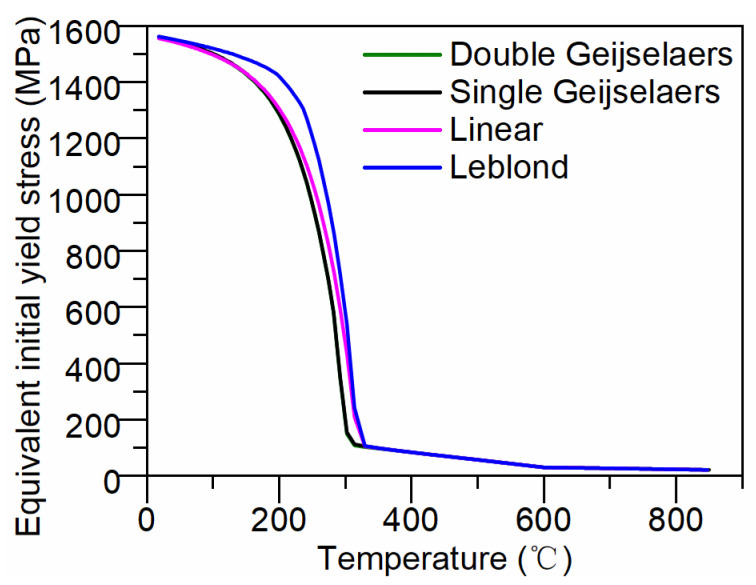
Effective initial yield strength on the surface of the cylinder under various weighting schemes.

To further investigate the reasons for differences in residual stress distribution, we calculated the evolution of the effective initial yield strength on the sample surface during the cooling process under various weighting schemes. The calculation results are illustrated in Figure 11. As depicted in Figure 11, during the initial quenching stage (850 °C to 585 °C), the cylinder microstructure consists of supercooled austenite (100% austenite), resulting in no variation in the effective initial yield strength across different weighting schemes. In the second stage of quenching (585 °C to 320 °C), supercooled austenite undergoes bainite transformation, causing a slight increase in the effective initial yield strength with decreasing temperature. Due to the minimal bainite formation on the surface, the effective initial yield strength of the four weighting schemes is very small, and the change in the effective initial yield strength remains low across all four weighting schemes, primarily influenced by temperature variations. During the third stage of quenching (320 °C to the end of cooling), supercooled austenite transforms into martensite, with approximately 97% martensite generated on the surface. The initial yield strength undergoes a highly nonlinear change due to the combined effects of temperature gradients and phase transformations. Significant differences are observed in the effective initial yield strength among the different weighting schemes during this cooling stage. The Leblond weighting scheme, which reduces the proportion of austenite yield strength, calculates the highest effective initial yield strength during martensite phase transformation. The linear weighting scheme, utilizing the austenite phase fraction ratio as the weighting factor, yields values intermediate between the Leblond and Geijselaers schemes. However, it still underestimates the material’s effective initial yield strength during phase transformation. Conversely, the Geijselaers weighting scheme seriously overestimates the proportion of austenite yield stress, resulting in lower effective initial yield strength calculations during phase transformation. Given the minimal bainite formation on the surface (approximately 3%), there is little difference between the effective initial yield strength calculated using single and double Geijselaers weighting schemes.

**Figure 12:**
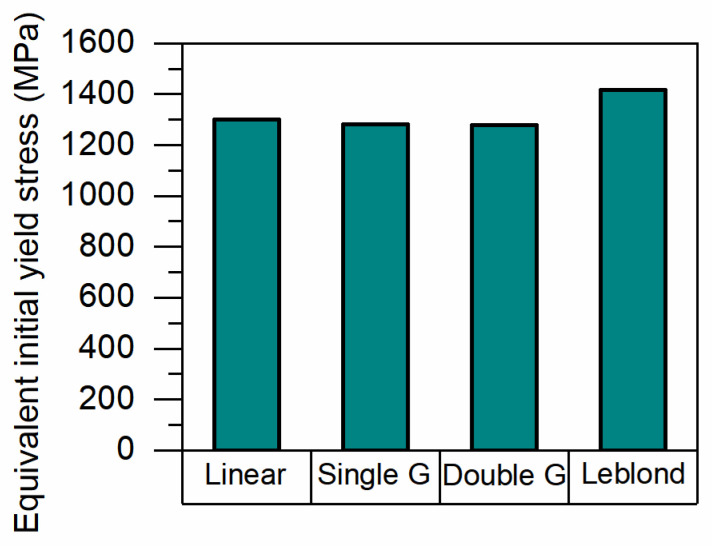
Equivalent initial yield stress of different weightings at 200 °C.

To more clearly illustrate the difference in initial yield strength caused by different weighting schemes, the equivalent initial yield stress at 200 °C is plotted separately in Figure 12. At this temperature, bainitic and martensitic transformations have occurred in the material. As shown in Figure 12, the equivalent initial yield stresses corresponding to linear weighting, single Geijselaers weighting, double Geijselaers weighting, and Leblond weighting are 1301 MPa, 1283 MPa, 1280 MPa, and 1417 MPa, respectively. These differences are the fundamental causes of the varying final residual stresses calculation.

**Figure 13:**
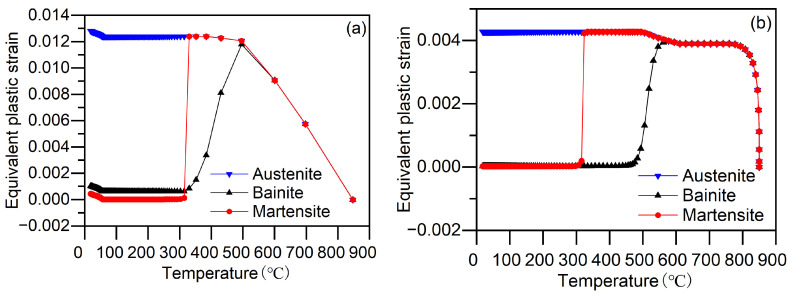
Equivalent plastic strain of each phase during quenching: (**a**) Surface; (**b**) Core.

To further investigate the plastic deformation occurring during quenching, we calculated the equivalent plastic strains of austenite, bainite and martensite phases, respectively, and compared them with the total equivalent plastic strain of the material. The results are depicted in Figure 13. The initial yield strength weighting scheme employed is the Leblond weighting scheme. Figure 13a,b illustrate the equivalent plastic strains of each phase on the surface and core of the cylinder, respectively. Figure 13a,b clearly show that the predominant equivalent plastic strain in the cylinder occurs in the austenite phase. During the initial stages of quenching, rapid cooling creates significant temperature gradients, resulting in high thermal stress. Due to the low yield strength of high-temperature austenite, plastic deformation occurs in austenite. During the bainite transformation stage, the face-centered cubic austenite transforms into body-centered cubic bainite, causing volume expansion. Austenite also undergoes plastic deformation to accommodate this volume change induced by the phase transformation. In the martensite transformation stage, the extent of austenite’s plastic deformation is less than expected, primarily because martensite phase transformation occurs at lower temperatures where the yield strength of austenite is enhanced. Furthermore, Figure 13a,b indicate relatively lower equivalent plastic strains for bainite and martensite. At the end of quenching, the equivalent plastic strains for bainite and martensite on the surface are 0.00104 and 4.459 × 10^−4^, respectively, while in the core they are 3.035 × 10^−5^ and 2.499 × 10^−5^, respectively. The yield strength of bainite and martensite is significantly higher compared to austenite. Further research is necessary to ascertain whether the equivalent plastic strain observed in bainite and martensite originates from austenite’s equivalent plastic strain or from the complex stress state during quenching surpassing their yield strengths.

## 4. Conclusions

Finite element models were developed with four different initial yield strength weighting schemes to simulate the quenching process of AISI 4140 cylinders. The predicted residual stress results for each weighting scheme were compared with experimental data. The study shows that the model with Leblond weighting provides the most accurate prediction of residual stress, making it a valuable tool for optimizing the quenching process through finite element simulation. The main conclusions are as follows:

1. Different initial yield strength weighting schemes influence the equivalent initial yield strength by altering the proportions of austenite yield strength in the mixed-phase material.
2. While the weighting scheme has little impact on the location of maximum axial residual tensile and compressive stresses, it significantly affects their amplitudes.
3. The model with Leblond weighting results in the highest equivalent initial yield strength, followed by the Geijselaers and linear weighting schemes.
4. The finite element model with Leblond weighting more accurately predicts the residual stress distribution after quenching.
5. Finite element simulations indicate that plastic deformation during quenching is primarily caused by supercooled austenite, with only a minor contribution from bainite and martensite.

## Figures and Tables

**Table 1:** Chemical composition of AISI 4140 steel.

Elements	C	Si	Mn	Cr	Mo	Ni	Fe
Weight percent (%)	0.4	0.27	0.58	1.0	0.17	0.022	Balance

**Table 2:** Thermal expansion coefficient of each phase of AISI 4140.

Phase Composition	Austenite	Bainite	Martensite
TEC ^1^ (10^−5^ K^−1^)	2.25	1.3	1.15

^1^ TEC is abbreviation of thermal expansion coefficient.

**Table 3:** Normalized saturation function of TRIP.

Type	Expression
Abrassart	$\xi \left(3-2\sqrt{\xi }\right)$
Desalos	$\xi \left(2-\xi \right)$
Leblond	$\xi \left(1-\ln\left(\xi \right)\right)\;\mathrm{for}\;\xi >0.03,\;\mathrm{zero}\;\mathrm{otherwise}$
Tanaka	ξ

## Data Availability

The raw data supporting the conclusions of this article will be made available by the authors on request.

## References

[1] Eddahhaoui F.-Z., Najem A., Elhawary M., Boudalia M., Campos O.S., Tabyaoui M., José Garcia A., Bellaouchou A., Amin H.M.A. (2024). Experimental and Computational Aspects of Green Corrosion Inhibition for Low Carbon Steel in HCl Environment Using Extract of Chamaerops Humilis Fruit Waste. J. Alloys Compd..

[2] Boutoumit A., Elhawary M., Bellaouchou A., Boudalia M., Hammani O., José Garcia A., Amin H.M.A. (2024). Electrochemical, Structural and Thermodynamic Investigations of Methanolic Parsley Extract as a Green Corrosion Inhibitor for C37 Steel in HCl. Coatings.

[3] Zuo X.W., Chen N.L., Gao F., Rong Y.H. (2014). Development of Multi-Cycle Quenching–Partitioning–Tempering Process and Its Applications in Engineering. Int. Heat Treat. Surf. Eng..

[4] da Silva A.D., Pedrosa T.A., Gonzalez-Mendez J.L., Jiang X., Cetlin P.R., Altan T. (2012). Distortion in Quenching an AISI 4140 C-Ring—Predictions and Experiments. Mater. Des..

[5] Jung M., Kang M., Lee Y.-K. (2012). Finite-Element Simulation of Quenching Incorporating Improved Transformation Kinetics in a Plain Medium-Carbon Steel. Acta Mater..

[6] Esfahani A.K., Babaei M., Sarrami-Foroushani S. (2021). A Numerical Model Coupling Phase Transformation to Predict Microstructure Evolution and Residual Stress during Quenching of 1045 Steel. Math. Comput. Simul..

[7] O’ Brien E.C.H.C., Yeddu H.K. (2020). Multi-Length Scale Modeling of Carburization, Martensitic Microstructure Evolution and Fatigue Properties of Steel Gears. J. Mater. Sci. Technol..

[8] Asi O., Can A.Ç., Pineault J., Belassel M. (2009). The Effect of High Temperature Gas Carburizing on Bending Fatigue Strength of SAE 8620 Steel. Mater. Des..

[9] Liu Y., Qin S., Zhang J., Wang Y., Rong Y., Zuo X., Chen N. (2017). Influence of Transformation Plasticity on the Distribution of Internal Stress in Three Water-Quenched Cylinders. Metall. Mater. Trans. A.

[10] Park S., Kim D.-W., Kim J., Lee S.Y., Kwon D., Han H.N. (2020). A Finite Element Simulation for Induction Heat Treatment of Automotive Drive Shaft. ISIJ Int..

[11] Sugianto A., Narazaki M., Kogawara M., Kim S.Y., Kubota S. (2010). Distortion Analysis of Axial Contraction of Carburized-Quenched Helical Gear. J. Mater. Eng. Perform..

[12] Sugianto A., Narazaki M., Kogawara M., Shirayori A., Kim S.-Y., Kubota S. (2009). Numerical Simulation and Experimental Verification of Carburizing-Quenching Process of SCr420H Steel Helical Gear. J. Mater. Process. Technol..

[13] Lee S.-J. (2013). Comparison of Two Finite Element Simulation Codes Used to Model the Carburizing of Steel. Comp. Mater. Sci..

[14] Ferguson B.L., Li Z., Freborg A.M. (2005). Modeling Heat Treatment of Steel Parts. Comp. Mater. Sci..

[15] Hoang A.T., Phuong Nguyen X., Ibrahim Khalaf O., Xuan Tran T., Quang Chau M., Minh Hao Dong T., Nam Nguyen D. (2021). Thermodynamic Simulation on the Change in Phase for Carburizing Process. Comput. Mater. Contin..

[16] Kim D.-W., Cho H.-H., Lee W.-B., Cho K.T., Cho Y.-G., Kim S.-J., Han H.N. (2016). A Finite Element Simulation for Carburizing Heat Treatment of Automotive Gear Ring Incorporating Transformation Plasticity. Mater. Des..

[17] Zhong H., Wang Z., Gan J., Wang X., Yang Y., He J., Wei T., Qin X. (2020). Numerical Simulation of Martensitic Transformation Plasticity of 42CrMo Steel Based on Spot Continual Induction Hardening Model. Surf. Coat. Technol..

[18] Leblond J.B., Mottet G., Devaux J.C. (1986). A Theoretical and Numerical Approach to the Plastic Behaviour of Steels during Phase Transformations—I. Derivation of General Relations. J. Mech. Phys. Solids.

[19] Ju D., Zhang W.M., Zhang Y. (2006). Modeling and Experimental Verification of Martensitic Transformation Plastic Behavior in Carbon Steel for Quenching Process. Mater. Sci. Eng. A.

[20] Geijselaers H.J.M. (2003). Numerical Simulation of Stresses Due to Solid State Transformations: The Simulation of Laser Hardening. Ph.D. Thesis.

[21] Li J., Xu Y., Liu Y., He H. (2024). Investigation of Non-Uniformity of Temperature Distribution and Phase Transformation in Spiral Bevel Gears during Carburizing and Quenching. J. Mater. Sci..

[22] (2008). Non-Destructive Testing—Test Method for Residual Stress Analysis by X-Ray Diffraction.

[23] Kang D.T., Zhang H. (1983). Research on the distribution of residual stress along the cross-section of the workpiece in quenched and tempered large shafts. Trans. Met. Heat Treat..

[24] Leblond J.B., Mottet G., Devaux J., Devaux J.C. (1985). Mathematical Models of Anisothermal Phase Transformations in Steels, and Predicted Plastic Behaviour. Mater. Sci. Technol..

[25] Kakhki M.E., Kermanpur A., Golozar M.A. (2009). Numerical Simulation of Continuous Cooling of a Low Alloy Steel to Predict Microstructure and Hardness. Model. Simul. Mater. Sci. Eng..

[26] Voort G.F.V. (1991). Atlas of Time-Temperature Diagrams for Irons and Steels.

[27] Kirkaldy J.S., Venugopalan D., Marder A.R., Goldstein J.I. (1984). Phase Transformation in Ferrous Alloys.

[28] Lee Y.K. (2002). Effects of Nitrogen on γ →ε Martensitic Transformation and Damping Capacity of Fe-16%Mn-X%N Alloys. J. Mater. Sci. Lett..

[29] Fanfoni M., Tomellini M. (1998). The Johnson-Mehl-Avrami-Kohnogorov Model: A Brief Review. Il Nuovo C. D.

[30] Leblond J.B., Devaux J., Devaux J.C. (1989). Mathematical Modelling of Transformation Plasticity in Steels I: Case of Ideal-Plastic Phases. Int. J. Plast..

[31] Lee S.-J. (2012). A Kinetics Model for Martensite Transformation in Plain Carbon and Low-Alloyed Steels. Metall. Mater. Trans. A.

[32] Koistinen D.P., Marburger R.E. (1959). A general equation prescribing extend of austenite-martensite transformation in pure Fe-C alloys and plain carbon steels. Acta Metall..

[33] Denis S., Sjöström S., Simon A. (1987). Coupled Temperature, Stress, Phase Transformation Calculation. Metall. Mater. Trans. A.

[34] Houlsby G.T., Puzrin A.M. (2007). Principles of Hyperplasticity.

[35] Tong D., Gu J., Totten G.E. (2018). Numerical Investigation of Asynchronous Dual-Frequency Induction Hardening of Spur Gear. Int. J. Mech. Sci..

[36] Fasano A., Hömberg D., Panizzi L. (2009). A MATHEMATICAL MODEL FOR CASE HARDENING OF STEEL. Math. Models Methods Appl. Sci..

[37] Kang S.-H., Im Y.-T. (2007). Finite Element Investigation of Multi-Phase Transformation within Carburized Carbon Steel. J. Mater. Process. Tech..

[38] Fischer F.D., Reisner G., Werner E., Tanaka K., Cailletaud G., Antretter T. (2000). A New View on Transformation Induced Plasticity (TRIP). Int. J. Plast..

[39] Taleb L. (2001). Experimental Analysis of Transformation Plasticity. Int. J. Plast..

